# Supplementary material for: Are carbon emissions trading and green financial instruments synergistic? -Comprehensive quantitative research based on content analysis
Source: PLoS One. 2024 Mar 7;19(3):e0298601. doi: 10.1371/journal.pone.0298601 (PMC10919601; doi:10.1371/journal.pone.0298601)
Supplement: S1 Data — (ZIP) [file pone.0298601.s001.zip › Data/地级市Z_F工作报告.zip!/║■─╧/┬a╡╫╩╨/└┤╫╘╣1⁄2╓┌║┼í╛┬φ┐╦╩2╛▌═°í┐_2016.docx]

各位代表：

　　现在，我代表市人民政府向大会作政府工作报告，请予审查，并请各位政协委员和其他列席人员提出意见。

　　一、“十二五”时期和2015年工作回顾

　　过去五年，全市上下深入贯彻党的十八大和十八届三中、四中、五中全会精神，认真落实省委、省政府和市委的决策部署，主动适应经济发展新常态，大力实施融城、娄商返娄、对接大长沙战略，强力推进六个“四年行动计划”，加快“四化两型”建设步伐，总体完成了“十二五”确定的目标任务。

　　经济发展稳中有进，综合实力不断增强。地区生产总值、固定资产投资总额相继迈上千亿台阶，去年分别达到1291.38亿元、1108.39亿元，年均增长10.2%、28.6%。去年实现工业增加值593.38亿元、社会消费品零售总额433.82亿元，年均分别增长10.9%、14.8%。一般公共预算收入、地方收入分别达到95.37亿元、59.92亿元，年均增长11.1%、14.8%。银行机构本外币存款余额、贷款余额分别达到1285亿元、791亿元，年均增长14.4%、15%。全面小康总体实现程度达到83%。

　　结构调整步伐加快，转型发展取得实效。三次产业结构调整为14.7:51.3:34，第三产业提高2.5个百分点。工业内部结构逐步优化，六大高耗能行业增加值占比下降15.43%，高技术、高加工度和战略性新兴产业年均分别增长17.6%、18.3%、15.9%，娄底经开区晋级为国家级经济技术开发区，万宝新区列入长株潭城市群两型社会示范区，娄星工业园获批省级工业集中区。农业产业化加快推进，农产品加工产值达到156亿元，年均增长21%。现代金融、商贸物流、文化旅游产业快速发展。争取省政府出台了支持娄底转型发展的“9号文件”，为我市转型发展创造了更多机遇。

　　基础设施逐步改善，城乡面貌焕然一新。沪昆高铁、娄邵高铁相继通车，高速公路建成4条、开工3条，改造二级干线公路460公里，硬化农村公路9650公里，“井”字型高速公路网、“米”字型铁路网基本形成，长期制约发展的交通瓶颈得到有效破解。新型城镇化建设步伐加快，城镇化率达到43.8％。新农村示范建设扎实开展。完成了2048个村的电网改造、663座病险水库的除险加固和530处惠及128万农村居民的饮水工程。“十二五”主要污染物减排任务和湘江流域污染防治第一个“三年行动计划”全面完成，城市生活垃圾无害化处理率达到100%，县城以上城镇生活污水集中处理率达到89.04%，全市森林覆盖率达到50.21%。

　　改革开放深入推进，发展活力日益彰显。按时序要求完成了9大类112项改革任务。行政审批事项精简了73%，办理时限缩短50%以上。商事制度改革、营改增试点、地方金融改革稳妥推进。引进了4家股份制商业银行，2家企业在“新三板”成功挂牌。“湘博会”、“湖南（娄底）汽车板及汽车零部件产业合作对接会”正逐步成为我市的两个省级招商平台，五年实际利用内资、到位外资年均分别增长31.55%、17.83%，完成进出口总额79.15亿美元，是“十一五”的1.7倍。

　　社会事业协调发展，人民群众得到更多实惠。累计投入财政民生资金674.5亿元。整合扶贫资金17.2亿元，减少贫困人口54万。新增城镇就业19.33万人、农村劳动力转移就业28.97万人，城乡居民人均可支配收入分别从13274元、4442元提高到21838元、8655元。建设保障性住房12.44万套，改造农村危房4.16万户。新建农村幸福院435个，改扩建敬老院53个。新增学位6.5万个，建成农村合格学校994所。公立医院改革顺利推进，医疗服务和医保体系不断完善，城乡基本医疗保险参保率稳定在96%以上，城乡居民社会养老保险制度实现全覆盖。计生工作连续五年获全省先进。文化体育惠民工程扎实推进，现代公共文化服务体系加快构建。安全生产、信访维稳形势明显好转，进入全省先进市行列。

　　国防动员和后备力量建设扎实有为，军民融合务实推进，全民国防教育创新探索有效，双拥共建和参建参治效益显现，履行使命任务能力持续提升。法治政府建设取得明显成效，在全省率先出台《行政执法监督办法》。科技创新平台建设得到加强，组建国家级检测测试中心和工程技术研究中心产业化示范基地各1个，创新能力明显提升。食品药品监管体制进一步理顺。圆满完成第三次经济普查。国资监管体系逐步完善，央企对接合作取得成效。“数字娄底”地理空间框架建设全面完成，项目用地保障得到加强。人防、地方志、电子政务、住房公积金、档案、保密、地震、气象、外事侨务、民族宗教、对台、贸促、工会、共青团、妇女儿童、老龄、科协、红十字会、慈善、残联等各项工作均取得新的进步。

　　2015年，是“十二五”时期的重要组成部分，是极不平凡的一年。面对前所未有的压力，我们保持定力，坚定信心，在危机中抓机遇，在困难中谋发展，在逆势中稳增长，全年地区生产总值增长7.6%；一般公共预算收入、地方收入从9月份起开始实现正增长，扭转了连续8个月负增长的局面；固定资产投资增长18%；社会消费品零售总额增长10.8%；城乡居民人均可支配收入分别增长7.5%、10.5%。确保了全市经济运行缓中趋稳、稳中有升、逐步向好，确保了民生事业的持续改善，确保了社会大局的总体稳定，确保了“十二五”目标任务和六个“四年行动计划”的圆满收官。

　　2015年，我们突出抓了六个方面的工作：一是紧盯项目建设，用倒逼机制扩投资稳增长，助推了发展升温。去年5月底6月初和12月中下旬，分两次开展“重点项目集中开工、竣工”活动，“四个一批”重点项目开工率、竣工率分别达到99.2%、97.6%，分别提高5.3、4.8个百分点，营造了大抓项目建设的浓厚氛围，形成了项目集中上马的拉动效应。二是创新平台建设，强化了发展手段。组建兴娄融资担保公司、市创投集团、百通中小企业转贷资金公司等三个融资平台，从不同角度发力，有效缓解了项目建设融资难、融资贵问题。创新项目建设模式，推出了首批40个PPP项目，撬动社会资本131亿元。三是全力争取政策、对接政策，有效释放了政策红利。积极对接上级相关政策，主动把政策具体化、项目化。分四批争取了国家专项建设基金32.39亿元、申报发行企业债券36亿元。四是发扬啃“硬骨头”的精神，化解了一批难题。针对项目建设推进中多年积压的矛盾，开展了“集中会审，逐个解套”行动，使一些久拖不决的难题得到妥善化解；针对消费市场疲软问题，及时出台了19条促消费稳增长“办法”，扩大了有效需求；针对商品房“竣工扫尾难、库存消化难、交房办证难、税费清缴难”等问题，强力开展了房地产业“解难题、促发展、保稳定”专项行动，取得了初步成效；针对民间融资风险，采取系列综合措施，守住了不发生区域性、系统性金融风险的底线；针对非法超限超载严重的状况，创新模式强力“治超”，荣获全省先进。五是不遗余力改善民生，人民群众有了更多获得感。圆满完成为民办实事项目，完善社会保障体系，积极扩大就业，城乡居民人均可支配收入有新的增加。加大了对水府庙库区的保护与利用，与湘潭市签署了《共同保护水府庙行动宣言》，在顶层设计、绿色发展、环境保护、项目推进等方面达成了广泛共识。湘潭至娄底的天然气管道建成，实现了年底前中心城区居民用上天然气的目标。集中开展控违拆违、中心城区禁止燃放烟花爆竹与殡葬改革整治攻坚行动，取得了阶段性成效，特别是“禁燃”工作，受到了市民和回娄老乡们的普遍好评。六是加强政府自身建设，进一步提高了运转效率、务实水平和攻坚能力。把落实人大决定、做好人大评议整改和加强政治协商，作为政府的份内事抓实抓好，认真办理人大代表建议议案168件、政协委员提案178件。启动了领导干部自然资源资产离任审计试点工作，审计监督和保障作用得到较好发挥。深入践行“三严三实”，市政府制定出台“六条规定”，就如何提高办文办会质量、加强协调配合、务实推进工作、坚持问题导向、勇于破解难题等六个方面作出明确规定和要求，通过市政府班子的带头执行，政府系统作风建设有了明显转变。

　　推项目、搭平台，争政策、引资金，解难题、惠民生，保安全、促稳定，这一些实实在在的举措，推动了重点工作的落地生根，促进了复杂矛盾的有效化解，我们要继续坚持，并在实践中不断加以完善提高。

　　各位代表！

　　回顾“十二五”，我们倍感欣慰。全市上下克服困难，顶住压力，办成办好了一系列大事、难事和实事：长韶娄高速建成通车，娄底大道加快推进，“长株潭一小时经济圈”、“市域一小时经济圈”发展格局初步形成；成功创建国家园林城市、国家卫生城市，并获得创建全国文明城市提名；圆满承办了第十二届省运会和第九届残运会，并取得了金牌总数省运会第二、残运会第一的好成绩；圆满完成了六个“四年行动计划”，等等。这些成就的取得，是市委坚强领导、总揽全局的结果，是市人大、市政协、娄底军分区鼎力支持的结果，是全市上下团结一心、共同奋斗的结果。在此，我代表市人民政府，向全市人民，向各民主党派、人民团体，向驻娄人民解放军和武警、消防官兵、政法干警，向中央、省驻娄单位，向关心支持我市发展的各界人士，表示衷心感谢和崇高敬意！

　　各位代表！

　　回顾“十二五”，我们也清醒地看到了发展中存在的困难和问题：一是经济总量不大，综合实力不强，增速在全省排名靠后；二是传统产业动力不足，新兴产业比重偏小，支撑发展的大项目、好项目不多，产业集中度不高，去产能、去库存、调结构的难度很大；三是城市发展空间比较狭窄，城乡公共服务和社会保障水平不高，小康娄底建设、脱贫攻坚的任务很重；四是金融安全、生态安全、生产安全的隐患还较多，维护社会和谐稳定的压力大；五是一些干部的思想观念、精神状态、工作作风、能力素质难以适应新常态新形势的需要，廉政勤政和效能建设亟待加强，等等。这些问题的存在，既有历史原因，也有现实原因；既有客观原因，也有主观原因。对此，我们要有清醒的认识，要有足够的思想准备，要有工作预案。必须立足于“从我做起，从现在做起”，不推诿、不回避、不等待，找准原因，对症下药，以愚公移山的精神，逐个逐个地把问题解决好，逐个逐个地克服前进道路上的艰难险阻。

　　二、“十三五”时期主要目标和任务

　　“十三五”时期是实现第一个百年奋斗目标、全面建成小康社会的决胜阶段，是我市爬坡过坎、负重奋进的关键时期。放眼看全球、看全国、看全省，世界经济在深度调整中曲折复苏，我国仍处于可以大有作为的重要战略机遇期，我省对接融入“一带一路”、长江经济带等国家战略，发展前景广阔，我市的外部环境总体向好。

　　面对这样的大背景，我们在冷静地思考：今后五年，我们娄底如何正确定位、找准发展路径？如何尽快走出困境、渡过难关？如何在全省发展的大棋盘中体现“娄底责任”、“娄底力量”？于是，我们紧密结合娄底实际，对照全面建成小康社会各项目标任务，根据市委四届九次全会精神，编制了《娄底市国民经济和社会发展第十三个五年规划纲要（草案）》，现提请大会审查。

　　“十三五”全市经济社会发展总的要求是：高举中国特色社会主义伟大旗帜，全面贯彻党的十八大和十八届三中、四中、五中全会精神，以邓小平理论、“三个代表”重要思想、科学发展观为指导，深入贯彻习近平总书记系列重要讲话精神，以“四个全面”战略布局和创新、协调、绿色、开放、共享发展理念为统领，以提高发展质量和效益为中心，坚持“加速转型、奋力赶超”，促进“三量齐升”，推进“五化同步”发展，统筹推进经济建设、政治建设、文化建设、社会建设、生态文明建设，确保如期全面建成小康社会，建设幸福娄底。

　　“十三五”时期我市的发展目标是：地区生产总值年均增长8.5%，保持与全省同步，力争略高于全省平均水平；居民人均可支配收入年均增长10%；固定资产投资年均增长15%以上；一般公共预算收入年均增长9%以上；城镇登记失业率控制在4.6%以内。经济结构更加优化，融城发展加快推进，民生福祉普遍提高，生态环境持续改善，体制机制更加健全。努力将我市建成全国转型发展示范城市，打造成为大长沙的拓展区。

　　实现上述目标，必须突出抓好五个重点：

　　（一）坚持创新发展，增强发展内生动力。大力实施创新驱动战略，发挥科技创新在全面创新中的引领作用，推进“质量兴企、名牌强市”和重大科技创新工程，以创新塑造发展优势，确保全社会研发投入占地区生产总值比重达到2.3%以上。推进大众创业、万众创新，鼓励发展众创、众包、众扶、众筹新模式，让创新创业在湘中大地竞相迸发、充满活力。加大供给侧结构性改革力度，坚持供需两端发力，以供给创新释放消费潜力，确保社会消费品零售总额年均增长12%。深度对接“中国制造2025”湖南行动，实施“互联网+”行动计划，培育壮大高端装备、新材料、信息技术、生物健康、节能环保、移动互联网、文化创意、大数据、云计算等战略性新兴产业，构建多点支撑的产业体系。力争三次产业结构调整为10:48:42，高新技术产业增加值占地区生产总值比重达到15%。

　　（二）坚持协调发展，构筑平衡发展格局。大力实施融城战略，加快对外“融入”，主动对接大长沙；加强对内“融合”，着力打造以娄涟双主板块、冷新副板块为支撑，以周边卫星镇为支点的湘中城市群，力争城镇化率达到52%以上。建立健全城乡规划体系，做大做强县域经济，加强“美丽乡村”建设，促进城乡统筹发展。以创建全国文明城市为总抓手，构建现代公共文化服务体系，促进物质文明和精神文明协调发展，建设文化强市。积极支持国防建设和军事改革，促进军民融合深度发展，提高防空、防灾应急救援一体化建设水平。

　　（三）坚持绿色发展，建设美丽宜居娄底。健全生态文明制度体系，大力发展循环经济，严守资源消耗上限、环境质量底线和生态保护红线。加快推进“两型”社会建设，加强环境治理和生态保护，实施“天蓝”、“水清”、“地绿”工程，加快创建国家森林城市，打造绿色家园。确保中心城区及县城空气质量优良率达到85%以上，全市集中式饮用水源水质达标率100%，9个地表水水质监测断面水质达到Ⅲ类及以上，森林覆盖率达到50.25%，通过政府、社会、企业和群众的共同努力，让全市人民喝上干净水，呼吸到新鲜空气，听到鸟儿叫，经常看到“蓝蓝的天上白云飘”。

　　（四）坚持开放发展，提升对外开放水平。加快推进财税、金融、国资、农业农村和要素市场改革，主动对接和融入国家“一带一路”、省“一带一部”的新战略、新格局，把我市打造成为沪昆经济带的重要支点城市。发展壮大产业园区，加快公共保税仓库、海关与出入境检验检疫机构建设，打造立体口岸体系和“大通关”平台，提升开放功能与服务水平。坚持娄商返娄战略，在此基础上注重以娄商引外商，拓宽视野，大力引进国内国外各方面的投资商，大胆走出去，积极参与国际合作。坚持以引进项目为龙头，把引项目与引资金、引人才、引管理、引理念、引技术结合起来，以开放包容、合作共赢的理念，善待每一位客商，促成每一个项目。

　　（五）坚持共享发展，共创幸福美好生活。把脱贫攻坚作为头等大事和第一民生工程，扎实推进“六个精准”，坚持发展生产、改善公共服务、全民教育三措并举，加大投入，整合资源，实现全面脱贫。坚持普惠性、保基本、重公平，持续加强教育、就业、社保、卫生计生、文化、体育、环保、养老、自然灾害防御等工作，提升群众增收能力，改善生产生活条件，确保在大湘西板块率先全面建成小康社会。强化生产、生态、金融安全责任，完善立体化社会治安防控体系，加强社会综合治理，让全市人民更有安全感、更有获得感、更有幸福感！

　　各位代表！

　　未来五年，全面建成小康社会，是我们必须完成的总任务；建成全国转型发展示范城市、打造大长沙的拓展区，是我们的基本目标。“加速转型”，是我们娄底未来发展的必然选择，是不能错过的、“亡羊补牢”般的机遇；但是“转型”有个较长的阵痛期，不可能立竿见影，我们要有凤凰涅槃、浴火重生的顽强意志，保持战略定力。“奋力赶超”，是全市上下应有的精神风貌，既需要持续不断、久久为功的“奋力”；又需要全市上下，包括党政干部、人民群众和市场主体的共同“奋力”。既“赶超”自己，确保年年都有新发展，年年都有新进步；又“赶超”兄弟市州，力争进入全省第二方阵。“打造大长沙的拓展区”，不仅是一个定位问题，更是一种目标和要求；不仅要主动对接大长沙的产业发展，而且要学习大长沙的思想观念和进取精神。站在“十三五”的起点上，面临“转型”和“赶超”的双重压力，我们必须以无愧于时代的使命感和责任感，昂首挺胸，披荆斩棘，勇往直前，向着全面建成小康社会的伟大目标，一起出发！

　　三、2016年工作重点

　　今年我市经济社会发展的主要预期目标是：地区生产总值增长8%，一般公共预算收入、地方收入均增长5%，固定资产投资增长17%，社会消费品零售总额增长11%，进出口总额增长10%，城乡居民人均可支配收入分别增长8.5%、10.5%，节能降耗完成省定目标任务。

　　重点抓好七个方面的工作：

　　（一）着力推进产业转型升级，壮大实体经济

　　千方百计提振工业。工业兴，则百业兴；工业强，则财力强。因此，我们要下定决心，排除万难，大抓工业，提振工业。立足打造“娄底制造”目标，积极对接“中国制造2025”和“湖南制造强省五年行动计划”，强力推进湖南汽车产业配套基地、板材深加工产业基地和不锈钢产业基地等三大基地建设，优化提升农机、应急制造和电子陶瓷三大特色产业，重点抓好薄板深加工产业园、精细电子陶瓷产业基地的开工建设，推进星泰汽车零配件制造产业园、三一配套产业园等续建项目竣工投产，力争全年制造业产值达到600亿元以上。立足抢占未来产业制高点，大力培育和发展新材料、生物医药、新能源、电子信息、节能环保等战略性新兴产业，重点抓好住宅产业化基地、卫星导航车载终端平台等项目的开工建设，加快三泰新材轧辊扩建、文昌科技金属半固态成形生产线改扩建、金品电器智能电视生产线等续建项目进度，推进三迅新能源汽车动力电池、星珂照明、晶盛光伏电站等新建项目投产，确保全年新增高新技术企业7家以上，战略性新兴产业增加值增长15%以上。立足钢铁、有色、能源、建材、化工五大传统产业的“强链、补链、升级”，促进“两化”融合，抓实一批技改项目，催生一批产业增长点，力争技改投资增长10%以上。运用政府采购、扩大消费、支持企业拓展市场等方式，因企施策，帮助企业共度难关。重点支持涟钢、冷钢等企业逆势突围，帮助化工、煤炭、水泥等行业脱困发展。

　　大力发展现代服务业。做大做强商贸物流业，重点推进湘中国际物流园、诚通现代物流园、五江商贸城和县一级物流体系建设等续建项目，高铁南站总部基地、万宝新区电子商务集聚区等新建项目，加快全国中小商贸流通企业服务体系建设，构建现代物流体系。在中心城区和县市城区规划、建设一批特色街、名品店，培育“夜经济”，做活节庆经济、赛事经济和会展经济，有效激活消费、留住消费。加快发展文化旅游业，抓好仙女寨、水府庙文化旅游、湄江国家地质公园二期、曾国藩故里、大熊山国家森林公园基础设施以及中华蚩尤始祖文化园、梅山古镇保护与建设等项目，加快融入全省黄金旅游线路，打造生态文化旅游休闲目的地，争取全年旅游接待人次、旅游总收入均增长16%以上。大力发展健康养老产业，重点加快众一健康城、东部新区健康产业园、龙山与天门健康养生基地、青山养老健康城等项目建设。培育和发展现代金融产业，积极引进银行、保险、证券、期货等机构来娄设立分支机构，完成新化县和冷水江市设立村镇银行。加快发展电子商务，进一步推进冷水江电商产业园发展，支持新化县、双峰县创建电子商务进农村示范县。

　　加快发展特色高效农业。加强农村水利等基础建设和基本农田保护，推广先进适用农机，抓好粮食生产与收储工作，确保粮食安全。按照“做大规模、提升品质、培育特色、舞好龙头”的思路，加强资源整合，推进土地流转，优化种养结构，巩固提升粮油菜、畜禽水、果木竹等生产基地，创建国家、省级农业标准化示范基地，着力培育“三黑一菌”等特色品牌，加快一二三产业融合发展。支持涟源打造全省农产品深加工产业特色县，加快桥头河现代农业产业园创建国家、省级农业科技示范园。力争创建1家以上国家级农业产业化龙头企业并上市，新增2家以上销售收入过亿元的农业产业化龙头企业，培育5个省级以上名牌农产品，实现农业产业化龙头企业加工销售收入增长15%以上。

　　发展实体经济，需要一大批有责任担当、有战略眼光、有创新精神的企业家！我们要旗帜鲜明地尊重企业家、爱护企业家、精心培育企业家，充分发挥他们的示范引领作用。要大张旗鼓地激励大众创业、万众创新，着力创造公平竞争的市场环境，激化各类市场主体的创造活力。通过几年努力，让人们看到娄底大地上，不仅有一片片拔地而起的高楼大厦，还有一大批茁壮成长的、能支撑财税持续增长的纳税大户！有一大批顶呱呱的、响当当的、引领娄底实体经济发展的知名企业家！

　　（二）积极扩大有效投资，增强持续发展动能

　　突出重大项目引领。精心部署、组织开展“项目建设大比拼”活动，全年安排500个市级重点建设项目，实施60个省级重点建设项目，年度计划投资600亿元。确保100个重大项目竣工投产，200个项目开工建设，100个项目加快推进。结合“中国制造2025”、“互联网+”、生态文明、新型城镇化等重大战略实施，深度开发储备200个以上项目，切实积蓄发展后劲。

　　夯实园区发展平台。加快简政放权，逐步实现国家级园区享受市级审批管理权限、省级园区享受县级审批管理权限，着力形成“政府主导、业主开发、政企共建、项目先行”的园区运行模式。推进产城融合，加快“623工程”建设，促进标准厂房、基础设施与公共平台建设提质，改善园区软硬环境，提升园区的承载力。坚持注重特色、错位发展，抓好一批专业园区建设，引导和推动产业、企业、技术、人才向园区集聚，力争各省级以上园区至少引进2－3个总投资过10亿元的产业项目，引进160家创新创业企业，确保园区投资增长25%以上，园区规模工业增加值增长15%以上，园区主导产业产值占比达到70%以上。

　　强化各类要素保障。加强资金保障，积极立项争资，确保争取上级各类资金超过全省平均水平；强化战略招商引资，重点瞄准长三角、珠三角、京津冀、中关村等重点区域和央企外企，有针对性地开展小分队招商、专题招商和全端产业链招商，积极探索专业队伍的中介招商，力争内联引资增长18%以上；加强政银企对接，积极发展和运用私募债、股权投资、产业投资、创业投资等多元化融资方式，加大银行贷款和直接融资力度，支持娄星区湘村高科、高盛板业到“新三板”挂牌交易融资；推动PPP项目建设模式，带动更多民间投资。大力推进节约集约用地，盘活存量土地，提高单位面积投资强度和产出水平，为项目建设拓展空间。推进“两集中两到位”改革，规范流程，提高审批效率，严厉惩处“吃拿卡要”、阻工闹事和“三强”等违纪违法行为，切实优化发展环境。

　　（三）加快城乡统筹发展，努力建设美好家园

　　推进融城发展。加快对接融入大长沙，推进城市“东进”，完成水府庙库区保护利用总体规划编制，启动滨湖新城建设，规划建设娄水大道、环库公路及配套交通基础设施，拉近与大长沙的时空距离。以全省并乡合村工作为契机，稳步推进中心城区与周边乡镇融合发展，拓展中心城区发展空间。以娄底大道为主轴，加快构建融合发展的交通网络大格局，着力打造湘中城镇群，带动周边农村发展。

　　关于融城发展，一方面，要抓好交通融城、产业融城；另一方面，要尽快启动行政区划调整，统筹做好融城规划布局，推进区划融城。还有一个重要的任务是，要齐心协力推进“文化融城”，强化“娄底意识”，破除狭隘的地域观念。娄底，是448万娄底人民的娄底，是天下娄底人共同的娄底，在天南地北打拼的娄底儿女，在娄底辖区内工作、创业、学习、生活的所有人，都是娄底的一分子。归属娄底，认同娄底，热爱娄底，建设娄底，应成为我们的基本义务和责任！世界那么大，走到任何地方，如果有人问，你是哪里人？我们要自豪地说：我是湖南娄底人！

　　加强基础建设。强力推进益娄、娄衡、安邵等3条高速公路和娄底大道、G234等干线公路建设，继续推进G60醴娄扩容、龙琅、龙武等3条高速公路和S238新化温塘至金湾、S336娄星双江至涟源七星等6条干线公路的前期工作，全力抓好农村公路及配套设施建设，进一步改善交通条件，让群众出行更加便利。加快中心城区提质步伐，重点抓好环线和主次干道建设，开工建设神童湾大桥、秋蒲街大桥，拉通甘桂路、吉星路、娄星南路等断头路，抓好新星路等道路提质改造，启动城市地下综合管廊建设，加快海绵城市试点工作；推进三板组、廖家、大科等城中村改造；加快涟水河、孙水河两岸的路、堤、截污干管、风光带“四合一”工程建设和城市黑臭水治理，把孙水、涟水打造成宜居宜业宜乐的两条城市“彩带”；抓好高铁南站枢纽一体化项目后续工程、娄底市第三水厂、天然气利用工程等项目建设，提高公共供水水质和燃气普及率；加强城市防洪防涝防火设施建设；持续推进光纤入户工程，逐步实现城市公共区域免费WIFI全覆盖。支持县市城区错位发展、做大做强，完善市政设施与服务功能，增强辐射带动能力，启动龙湖新区、梅山新区规划建设。加强重点乡镇建设，因地制宜培育发展一批特色强镇。

　　提升管理水平。以创建全国文明城市为抓手，启动智慧城市建设，加强城市精细化管理。推动禁止燃放烟花爆竹和殡葬改革整治常态化。加强道路交通管理，加大治超治堵力度。启动国家森林城市创建工作，做好山水生态文章。加强生活垃圾、污水综合处理，抓好娄底经开区工业废水集中处理厂建设，做好第三污水处理厂建设前期相关工作。深入开展城乡环境卫生整洁行动，加强农村建房规划与管理，推进新农村建设和“美丽乡村”建设，改善人居环境。加大名镇、名村保护力度，留住历史文脉和文化遗产。

　　（四）深入推进改革开放，充分释放发展活力

　　加大供给侧结构性改革。坚持供需两端发力，认真落实“去产能、去库存、去杠杆、降成本、补短板”五大重点任务，充分发挥市场的主导作用和企业的创新精神与商业智慧，努力培育和形成新供给、新动力。加快农机、生物制药、电陶、矿机、农产品和锑品等出口基地建设，力争年内建好服务外包基地，支持一批外向型企业建立境外产业园区，拓展国际市场。将棚户区改造、保障房建设与商品房去库存结合起来，全面实施征地拆迁货币化安置或现有存量商品房安置，积极化解房地产库存。

　　加快行政管理体制改革。支持省直管县体制改革试点工作。改进外商服务模式，推行重大项目审批代理制和联合审批制。加大公共资源交易整合力度，争取今年6月底前将全市范围内的各类公共资源交易项目纳入市公共资源交易中心交易。积极推进镇改街道办事处、乡改镇、村改居工作，确保6月底前圆满完成合村工作。

　　深化经济体制改革。进一步完善国有资产监管体制，健全法人治理结构，稳妥推进国有企业发展混合所有制经济，加快建立现代企业制度。深化商事制度和供销合作社综合改革，继续做好“三证合一、一照一码”登记改革工作。健全国有自然资源资产管理体制，按要求完成编制自然资源资产负债表国家级试点工作，推进不动产登记制度改革，全面完成农村土地承包经营权确权登记颁证改革任务。开展农村承包土地经营权抵押贷款试点，完成“一县两行”农村金融改革。试行科技专项资金后补助模式。推进“营改增”、全口径政府预算管理等财税体制改革。落实各项政策，大力推进创新驱动，健全创新创业体系，加快科技成果转移转化，促进科技与经济深度融合。

　　（五）坚决打好扶贫攻坚战，补齐小康社会建设的短板

　　坚持专项扶贫、行业扶贫、社会扶贫“三位一体”，以新化、涟源、双峰等扶贫开发重点县和574个贫困村为主战场，大力实施群众增收、异地搬迁、素质提升、兜底保障、基础扶贫等五大工程。做好移民安置和后期扶持工作，加快贫困移民脱贫解困。加强贫困农民培训，因村因户、分类指导实施产业扶持项目，助推金融精准扶贫。整合项目资金向贫困村、贫困户倾斜，加快实现水、电、路、气、房、环境治理“六到农家”，扎实做好就医、就学、养老、低保、五保、村级集体经济发展“六个落实”，力争贫困地区农民可支配收入增长20％，全年减少贫困人口9万人。让全市30多万贫困人口的生活好起来，是我们心中的牵挂，也是我们应尽的责任！

　　（六）切实筑牢安全底线，确保和谐稳定发展

　　夯实安全生产基础。全面落实安全生产“党政同责、一岗双责、失职追责”的要求，推进安全生产监管网格化，强化政府、行业主管部门安全监管责任和企业安全生产主体责任。深入推进安全生产标准化工作向规模以下工贸企业延伸和覆盖，力争上半年完成规模企业达标创建。强化重大动物疫病防控和农产品质量安全监管与检验检测，落实食品安全战略，强化食品安全监管，加快建设食品药品实验室，全面落实“四个最严”要求，让老百姓吃上放心食品、用上安全药品。继续开展“特种设备安全保障行”活动，加强危险化学品、油气管道、烟花爆竹等专项整治，抓好煤矿、非煤矿山、道路交通、建筑施工、校园、校车等重点领域的安全工作，坚决防止发生重特大安全事故。

　　建立生态安全屏障。推行领导干部自然资源资产离任审计制度和环境损害追究制度，促进生态协调发展。加快锡矿山地区、涟钢周边和独立工矿区生态修复和治理，扎实推进湘江保护和治理第二个“三年行动计划”。加强大气污染控制和治理，推进工业企业污染技术改造与深度治理，在重点行业实行强制性循环水利用技术改造，达到零排放。实行最严格水资源管理制度，加强饮用水源和湿地保护，提高水土保持和水源地保护能力。加强造林绿化、森林防火和森林资源保护，提高森林覆盖率，努力构建天蓝水清地绿的生态屏障。

　　防范金融安全风险。按照“分类要准、帮扶要实、处置要快、打击要严”的要求，稳妥推进民间融资风险分类处置工作，规范民间借贷行为，加强互联网金融风险防控，严厉打击非法融资、非法催债、高利借贷，坚决守住不发生系统性、区域性金融风险的安全底线。落实金融支持工业稳增长调结构增效益的若干意见，争取金融机构扩大贷款规模，增强金融服务能力，助推实体经济渡难关。重构社会诚信体系，坚决打击各类失信背信行为，依法维护金融债权，帮助金融机构化解不良资产，降低不良率。

　　（七）办好惠民利民实事，加强社会建设

　　积极扩大就业。全面落实就业创业政策，扎实做好高校毕业生、农村转移劳动力、就业困难人员等重点群体就业工作，确保城镇登记失业率控制在4.5%以内，新增城镇就业3.6万人，新增农村劳动力转移就业3.5万人。

　　发展社会事业。继续化解城区大班额，进一步改善农村办学条件，落实提升教师待遇政策和乡村教师支持计划，推动义务教育均衡发展；加强普惠性学前教育，推动高中、职业、民办、特殊教育协调发展，积极为湖南人文科技学院和娄底职业技术学院创造良好的发展环境。加快构建公共文化服务体系，完善县市区文化馆、图书馆及公共体育设施，推动乡镇、街道综合文化体育服务中心建设，加强文化市场、广电安全监管。完成市国家综合档案馆主体工程建设。深化医药卫生体制改革，鼓励社会办医，支持全科医生培养基地、市县两级妇幼保健院及精神病专科医院建设、综合医院重症医学科、儿科等项目建设，加强医疗、疾控、卫生应急体系建设，提升中医药服务能力。加快构建社会养老服务体系。坚持计划生育基本国策，完善计划生育目标管理责任制，大力推进人口网格化管理，落实全面两孩政策。加强统计基层基础工作，认真做好第三次农业普查。加强红十字会工作，大力发展慈善事业。

　　完善保障体系。探索实施五险统一征缴，强化社保扩面征缴。实施城乡居民大病保险制度，完善工伤保险浮动费率操作办法，扩大失业保险基金支出范围。完善被征地农民社会保障政策。扩大住房公积金覆盖面，加快住房公积金制度向全体农民工覆盖。落实大病救助政策。继续做好低收入家庭人员的生活救助和失能特困老人的保障工作。

　　创新社会治理。围绕创建“全国社会管理综合治理先进市”，强化源头预防，推进社会稳定风险评估，做好网络秩序和信息风险防范工作。推进“平安娄底”、“天网工程”建设，健全立体化社会治安防控体系，强化火灾隐患和消防违法行为综合治理，依法严厉打击严重暴恐犯罪、“两抢一盗”、传销等违法犯罪行为。全民动员，向毒品宣战，把禁毒工作抓出实效。注重运用法治思维和法治方式解决信访突出问题。加快建设覆盖城乡的公共法律服务体系，建立基层维稳与应急机制，全面排查化解矛盾纠纷，探索“费随事转、事费配套”方式，推进城乡社区治理创新。做好人防、地方志、电子政务、档案、保密、地震、气象、外事侨务、民族宗教、对台、贸促、工会、共青团、妇女儿童、老龄、科协、残联等各项工作。

　　提高精神文明和民主法制建设水平。全面实施公民道德建设工程，积极培育践行社会主义核心价值观，深化各类群众性精神文明创建活动，扎实推进创建全国文明城市各项工作。启动“七五”普法，增强全社会尊法学法守法用法观念。认真执行人大及其常委会的决议决定，主动接受人大的法律监督、工作监督和政协的民主监督，务实办理人大代表建议、议案和政协委员提案。严格依法行政，落实“三重一大”等民主决策流程和政府法律顾问制度，建立重大决策终身责任追究制度和责任倒查机制，提高依法决策、科学决策、民主决策水平。落实政府权力清单制度，大力推进政务公开，推广电子政务和网上办事，加快建设法治政府。

　　推进国防建设。加强国防后备力量建设，推动军民融合深度发展，巩固双拥共建成果，做好退役军人转业安置工作，加强民兵基层建设，促进经济建设和国防建设协调发展。

　　各位代表！做好今年各项工作，全面完成“十三五”目标任务，如期全面建成小康社会，必须切实加强政府自身建设。

　　加强政府自身建设，要求全市各级政府和全体政府工作人员，务必增强政治意识、大局意识、核心意识、看齐意识，不折不扣地贯彻执行中央、省委、省政府和市委的重大决策部署；务必践行“三严三实”要求；务必认真落实党风廉政建设的各项规定。在此基础上，要特别注重勤政务实，并努力做到以下四点：

　　第一，雷厉风行。政府的执行力，首先体现的是一个“快”字。定了的事情，就要快速行动，立说立行，不等不拖，抢抓时间，抢抓机遇，让项目早落地、早见效；让矛盾早化解、早清零；让群众早受益、早得实惠、早解忧愁。

　　第二，锲而不舍。经济社会发展中的各项工作，都会有程度不同的困难，这就需要锲而不舍，需要毅力和恒心。面对困难和矛盾，不退缩，不泄气，不虎头蛇尾。积极想办法，主动找“钥匙”，咬定目标不放松，以钉钉子和啃“硬骨头”的精神，持之以恒地抓到位，不达目的不罢休。

　　第三，精益求精。无论是日常的办文、办会、办事，还是抓改革、促发展、保稳定，都要注重规范，注重细节，注重精细化操作。不能只求“过得去”，而必须“过得硬”，力求高标准、高质量、高水平，把大事做圆满，把小事做精致，把难事做稳妥。

　　第四，任劳任怨。要崇尚爱岗敬业，吃苦不叫苦，有功不邀功，勤勤恳恳、心无旁骛地把每项工作干好。不张扬，不抱怨，也不怕他人埋怨。用快乐的心情对待工作，工作就是一种享受，而不是一种负担。把自己开成一朵最美丽的鲜花，既可以让他人赏心悦目，又可以让自己神清气爽、乐在其中。

　　各位代表！

　　展望“十三五”，我们心中有信念、有激情，我们肩上有使命、有责任！在新的历史起点上，让我们紧密团结在以习近平同志为总书记的党中央周围，在省委、省政府和市委的坚强领导下，深入贯彻“五大理念”，加速转型、奋力赶超，锐意进取、攻坚克难，为全面建成小康社会、建设幸福娄底而努力奋斗！
